# Upregulation of TIMM8A is correlated with prognosis and immune regulation in BC

**DOI:** 10.3389/fonc.2022.922178

**Published:** 2022-09-29

**Authors:** Yu Zhang, Lin Lin, Yunfei Wu, Pingping Bing, Jun Zhou, Wei Yu

**Affiliations:** ^1^ Endoscopy Center, Clinical Oncology School of Fujian Medical University, Fujian Cancer Hospital, Fuzhou, China; ^2^ Department of Medical Oncology, Clinical Oncology School of Fujian Medical University, Fujian Cancer Hospital, Fuzhou, China; ^3^ Department of Thoracic Surgery, The Affiliated Hospital of Southwest Medical University, Luzhou, China; ^4^ Academician Workstation, Changsha Medical University, Changsha, China; ^5^ Department of Clinical Pharmacy, Clinical Oncology School of Fujian Medical University, Fujian Cancer Hospital, Fuzhou, China

**Keywords:** Breast cancer, TIMM8A, biomarkers, prognosis, immune infiltration

## Abstract

**Backgrounds:**

Breast cancer is a common malignant tumors in women. TIMM8A was up-regulated in different cancers. The aim of this work was to clarify the value of TIMM8A in the diagnosis, prognosis of Breast Cancer (BC), and its association with immune cells and immune detection points. Gene mutations.

**Methods:**

The transcription and expression profile of TIMM8A between BC and normal tissues was downloaded from The Cancer Genome atlas (TCGA). The expression of TIMM8A protein was evaluated by human protein map. The correlation between TIMM8A and clinical features was analyzed using the R package to establish a ROC diagnostic curve. cBioPortal and MethSurv were used to identify gene alterations and DNA methylation and their effects on prognosis. The tumor immune estimation resource (TIMER) database and tumor immune system interaction database (TISIDB) database were used to determine the relationship between TIMM8A gene expression levels and immune infiltration. The CTD database was used to predict related drugs that inhibit TIMM8A, and the PubChem database was used to determine the molecular structure of potentially effective drug small molecules.

**Results:**

The expression of TIMM8A in breast cancer tissues was significantly higher than that in normally adjacent tissues to cancer. ROC curve analysis showed that the AUC value of TIMM8A was 0.679. Kaplan-Meier method showed that patients with high TIMM8A had a lower prognosis (Overall Survival HR = 1.83 (1.31 − 2.54), P < 0.001) than patients with low TIMM8A expression of breast cancer (148.5 months vs. 115.4 months, P < 0.001). Methylation levels at seven CpG were associated with prognosis. Correlation analysis showed that TIMM8A expression was associated with tumor immune cell infiltration. There was a significant positive correlation of TIMM8A with PDL-1, and CTLA-4 in BC. In addition, CTD database analysis identified 15 small molecular drugs that target TIMM8A, such as Cyclosporine, Leflunomide, and Tretinoin, which might be effective therapies for targeted inhibition of TIMM8A.

**Conclusion:**

In breast cancer, up-regulated TIMM 8A was significantly related to lower survival rate and higher immune invasiveness. Our research showed that TIMM 8A could be used as a biomarker for poor prognosis of breast cancer and a potential target of immunotherapy.

## Introduction

Breast cancer (BC) in women had exceeded lung cancer to be the most prevalent cancer, with an approximated 2.26 million new cases (11.7%) (1). BC was also the dominant reason of cancer death among women worldwide (2). Fortunately, the level of diagnosis and treatment has been continuously improved in recent years, and the mortality of BC has been continuously r decreased. However, the recurrence and metastasis still occur in patients within 5 years, and distal metastasis is a major reason of death in BC patients (3). Researches have exhibited that the prognosis of BC is affected by many clinical aspects, such as age, tumor size, histological grade, lymphatic invasion, lymph node, hormone receptor status, etc. However, as the molecular mechanism behind the invasive characteristics of BC is still unclear, it’s crucial to study new biomarkers for forecasting the prognosis of patients and tumor invasiveness. Establishment of a new risk prediction model for breast cancer would promote the medication and prognosis of BC patients (4, 5).

There is expanding studies that the immune system plays an important role in cancer (6). The tumor immune microenvironment (TIME) is a cellular immune ecosystem composed of immune cells (7), the extracellular matrix, fibroblasts, endothelial cells, and various cytokines. Continuing studies have shown that tyrosine kinase inhibitors (TKIs), immune checkpoint inhibitors (ICIs), and molecularly targeted drugs can effectively inhibit BC. Due to the highly dynamic TIME in BC (8), as well as glucose and lipid metabolism, BC may be associated with different types of medicine resistance to TKIs and ICIs.

TIMM8A (translocate of inner mitochondrial membrane 8a) has been found that TIMM8A is related to Mohr-Tranebjaerg syndrome and focal muscle Zhang Li disorder (9).The molecular functions include mitochondrial protein input and protein metabolism.TIMM8B is an important homologue of this gene. It has been reported that Mutations in the TIMM8A gene lead to Mohr-Tranebjrg Syndrome (MTS) (10), and TIMM8A is related to the proliferation of ovarian cancer cell line SKOV3/DDP subcutaneous xenografts (11).

However, the link between TIMM8A and BC has not been described. We presumed that TIMM8A levels were related to BC survival. Immune evasion mechanisms for BC adaptation include downregulation of antigen presentation/recognition, shortage of immune effector cells, blockage of maturation of anti-tumor immune cells, aggregation of immunosuppressive cells, creation of inhibitory cytokines, and upregulation of immune checkpoint modulators. The TME refers to the episode, growth and metastasis of tumor, which is closely related to the internal and external environment of tumor cells.

In this study, we found that the mRNA level of TIMM8A was up-regulated in BC. The up-regulation of TIMM8A is negatively related to clinical features. The diagnostic and prognostic role of TIMM8A in breast cancer was further evaluated. Here, we also analyzed the relationship between TIMM8A and immune cells, biomarkers and immune checkpoints (IC). The gene mutation, DNA methylation and related pathways associated with the episode and development of BC were also examined. In addition, the correlation between TIMM8A and immune infiltration was examined by Tumor Immune Estimation Resource (TIMER) and Tumor Immune System Interaction Database (TISIDB). Meanwhile, the relationship between TIMM8A and Important immune subsystem was evaluated. Our results could potentially reveal new targets and strategies for BC diagnosis and medication.

## Materials and methods

### TCGA data set

From the official website of TCGA (12) Link to download the transcriptional expression data of TIMM8A and the corresponding clinical information. We analyzed 33 registered cancers. Finally, for further investigation, we downloaded TCGA data, and we converted the RNA sequencing data from FPKM to TPM format and transformed log2. All data were downloaded from TCGA. This study did not require ethics committee approval.

### RNA sequencing data of TIMM8A in breast cancer

RNA-seq expression data of TIMM8A in breast cancer were also downloaded from TCGA and the XIANTAO platform (https://www.xiantao.love/). Therefore, after eliminating the invalid clinical data, the data of 1109 cases of breast cancer and 113 cases of normal tissues adjacent to cancer were kept. TIMM8A gene expression data and relevant clinical information including age, T-phase, N-phase, M-phase, tumor site, ER/PR/HER2 status were selected for inclusion. The mRNA expression data were X ± SD mean.

### Mutation and immune infiltration analysis

We used cBioPortal (http://www.cbioportal.org/) to analyze the mutation frequency of TIMM8A in BC. The type of mutation in TIMM8A in BC was further assessed using the Cancer Somatic Mutation Catalog (COSMIC) database (http://cancer.sanger.ac.uk). The correlation between TIMM8A expression and immune infiltration was assessed using the TIMER database (https://cistrome.shinyapps.io/timer/).

### The Human Protein Atlas (HPA)

HPA (https://proteinatlas.org/) contains human gene expression profile information of protein levels in normal tissues and tumor tissues (13). In this study, we compared the expression of TIMM8A protein in normal breast tissues and breast cancer tissues by HPA.

### GO/KEGG

Visualization using the ClusterProfiler package and the GGploT2 package analyzed the Kyoto gene and genomic Baike Encyclopedia (KEGG) pathway enriched by gene ontology (GO) and co-expression genes.

### GSEA function and pathway analysis

Gene set enrichment analysis (GSEA) was performed using the ggplot2 R package (V3.3.3) to demonstrate important functions and pathways between the two groups. Expression levels of TIMM8A were used as phenotypic markers. Adjusted p-values < 0.05, enrichment of normalized scores (|NES|) < 1, and false discovery rate (FDR) < 0.25 were significantly different.

### Construction and evaluation of nomogram

Personal predictions of 1-,3-and 5-year survival probabilities. Based upon the results of multivariate analysis, a histogram was constructed. The RMS R package (version 6.2-0) was used to generate Nomograms with important clinical features and calibration maps. Concordance indices and modified curves were used to estimate their predictive power.

### DNA methylation information of TIMM8A

MethSurv database (https://biit.cs.ut.ee/methsurv/) was used to evaluate DNA methylation of TIMM8A in TCGA database. And the prognostic value of CpG methylation in TIMM8A was analyzed.

### Tumor Immune Estimation Resource (TIMER) database

TIMER (https://cistrome.shinyapps.io/timer/) is a comprehensive online database analyzing various cancer types associated with immune penetration. We used the TIMER database to determine the relationship between TIMM8A expression and six types of immune infiltrates (B cells, CD4+ T cells, CD8+ T cells, neutrophils, macrophages, and dendritic cells).

### Tumor-Immune System Interaction Database (TISIDB)

TISIDB (http://cis.hku.hk/TISIDB/) was an online portal for tumor-immune system interactions. In this study, we used TISIDB to determine TIMM8A and tumor-infiltrating lymphocyte (TILs) expression in human cancers. The relative abundance of TILs was derived from gene expression profiles and gene set variation analysis. Spearman’s test was used to determine the correlation between TIMM8A and TILs.

### Immune checkpoints analysis

Correlation analysis between TIMM8A and immune checkpoints using the xiantao platform (www.xiantao.love) and the TIMER (https://cistrome.shinyapps.io/timer/) was analyzed by GGploT2 R package based on the expression of TCGA. Results with the P < 0.05 was considered statistically significant.

### Screening of small molecule therapeutic drugs

The CTD database was applied to predict potential associations of TIMM8A with drugs. CTD database (https://ctdbase.org/), can be used for research based on the relationship between chemistry, genes, phenotypes, disease and environment, promoted the understanding of chemical drugs and human health. Use the PubChem database (https://pubchem.ncbi.nlm.nih.gov/) to view the molecular structures of related drugs.

### Statistical analysis

All statistical analyses used R (V 3.6.3). Paired t-test and Mann-Whitney U test were used to determine differences between breast cancer tissue and adjacent normal tissue. Visualization was performed using the R package GGplot2 (14), the clusterProfiler package (version 3.14.3) (for GSEA analysis). ROC curve plotting was performed using the pROC package (15).

## Results

### The expression of TIMM8A at the pan-cancer level

**Figure 1:**
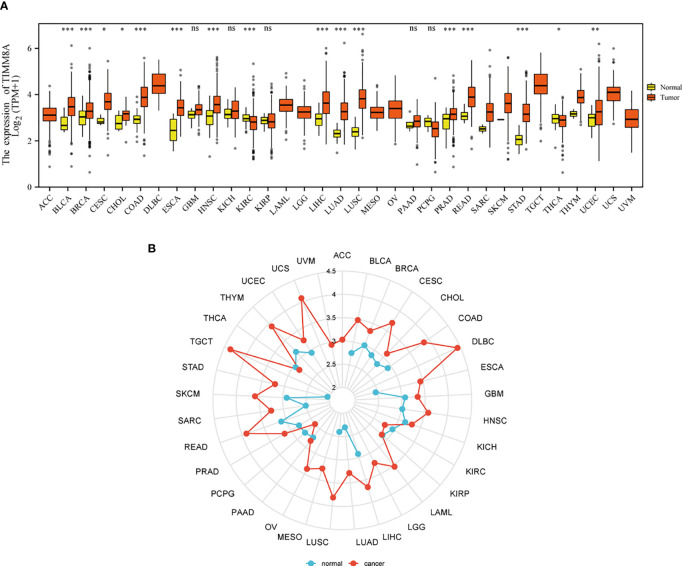
Expression patterns of TIMM8A from a pan-cancer perspective. Compared with normal tissues, TIMM8A mRNA expression was significant in 16 of 33 cancers. **(A)** Box plot; **(B)** Radar chart. (ns, p≥0.05; *p<0.05; **p<0.01; ***p<0.001).

**Figures 1A, B** show the mRNA expression of TIMM8A in different cancer types. The results suggest that compared with normal tissues, TIMM8A was significantly up-regulated in 16 of 33 cancers. It indicated that the expression of TIMM8A mRNA was abnormal in different cancer types.

### mRNA expression of TIMM8 in BC

**Figure 2:**
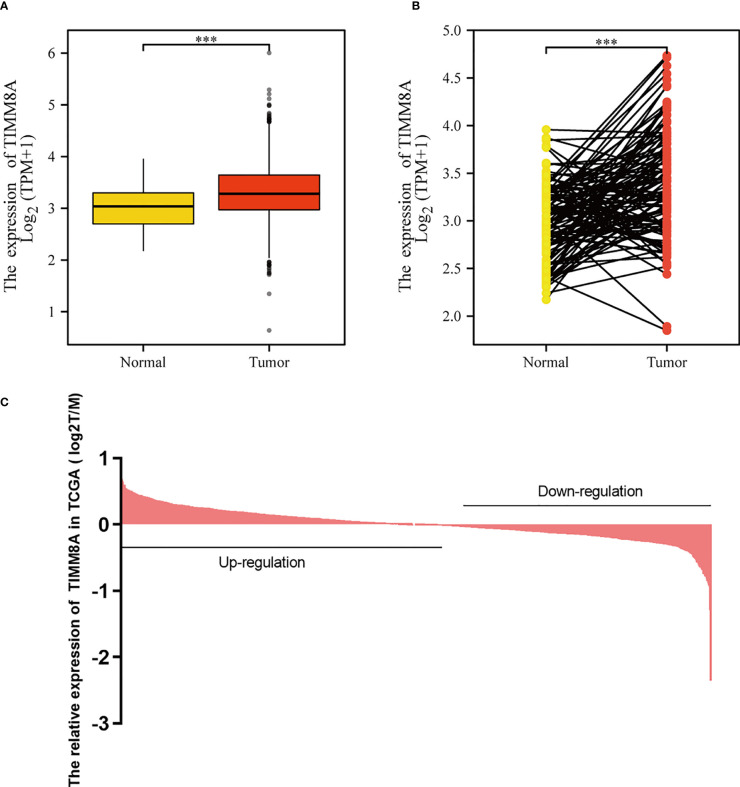
TIMM8A expression in TCGA. **(A)** TIMM8A mRNA expression levels in Tumor and Normal. **(B)** TIMM8A mRNA expression levels in 112 breast cancer patients and matched adjacent normal samples. **(C)** TIMM8A in TCGA expression in breast cancer tissue. (***p<0.001).

To examine the mRNA and protein expression of TIMM8A in breast cancer, we analyzed the expression data of TIMM8A in TCGA (**Figure 2C**). As shown in **Figure 2A**, the unpaired data analysis showed that the mRNA expression level of TIMM8A in breast cancer tissues (n = 1109) was significantly higher than that in normal tissues (n = 113) (**Figure 2A**, 3.391 soil 0.636 vs 3.005 soil 0.618, Mann -Whitney U-test, P < 0.001). Paired data analysis also revealed that TIMM8A messenger RNA expression levels were significantly higher in breast cancer tissues (n = 112) than in adjacent normal tissues (n = 112) (**Figure 2B**, 3.351 soil 0.597 vs 2.998 soil 0.617, P < 0.001). These results suggested that the mRNA expression of TIMM8A was upregulated in breast cancer tissues.

### Protein expression and diagnostic and prognostic value

**Figure 3:**
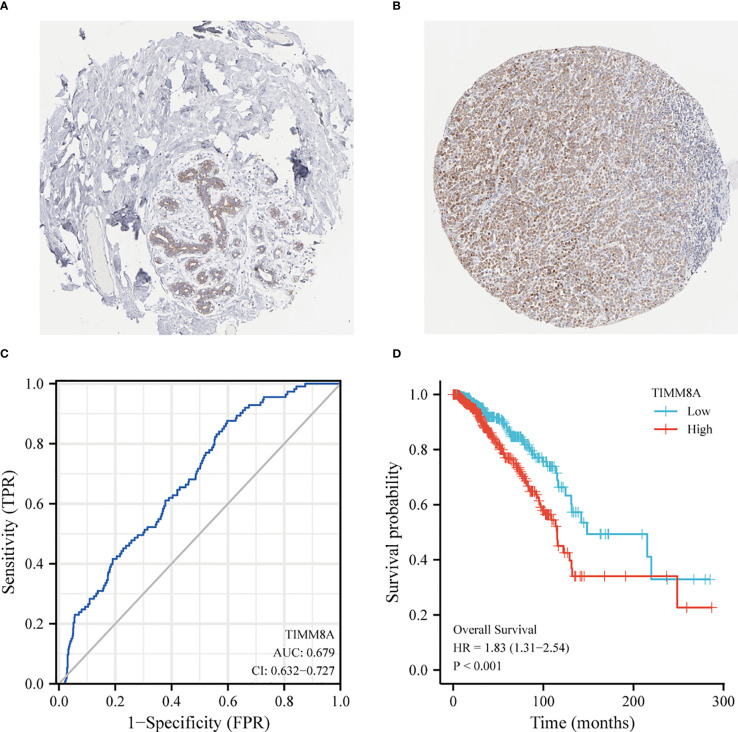
Protein Expression and Diagnostic and prognostic value of TIMM8A. **(A)** TIMM8A protein levels based on Human Protein Atlas. Normal tissue; **(B)** Tumor tissue. **(C)** The ROC curve shows the AUC value of TIMM8A. **(D)** The Kaplan-Meier survival curve shows the OS of breast cancer patients.

The HPA immunohistochemical staining database was used to show that TIMM8A protein expression was up-regulated in breast cancer tissues (**Figures 3A, B**). ROC curve analysis was applied to investigate the diagnostic value of TIMM8A in differentiating breast cancer from normal breast cancer. ROC curve analysis showed that the AUC value of TIMM8A was 0.679, CI = 0.0.632-0.727 (**Figure 3C**). To explore the relationship between TIMM8A expression and OS in breast cancer patients, we drew Kaplan Meier curves. The OS of patients with breast cancer with high TIMM8A expression was shorter than that of patients with breast cancer with low TIMM8A expression (115.4 months vs. 148.5 months, P < 0.001) (**Figure 3D**).

### Clinicopathological characteristics

**Figure 4:**
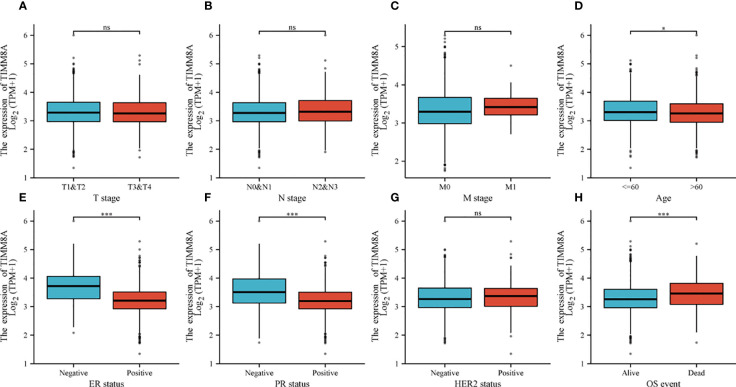
The relationship between TIMM8A mRNA level and clinicopathological characteristics. TIMM8A mRNA expression is negatively correlated with age **(D)**, ER **(E)** and PR **(F)** status, positively correlated with OS event **(H)** and there is no significant difference in T **(A)**, N **(B)**, M **(C)** levels, and HER2 **(G)** status. (ns, p ≥ 0.05; *p < 0.05; ***p < 0.001).

To explore the relationship between TIMM8A mRNA expression and clinicopathological features in breast cancer tissues, we performed Mann-Whitney U test and logistic regression analysis. As shown in **Table 1** and **Figures 4A–H**, there was a significant difference in different T phase (P = 0.016), PR status (P < 0.001), ER status (P < 0.001), HER2 status (P = 0.014), and OS event (P < 0.001). However, the expression level of TIMM8A was not correlated with N phase (P = 0.455), M phase (P = 0.162), age (P = 0.120). In summary, these results indicated that TIMM8A was related to hormone levels, age, T phase, os events, and further suggested that TIMM8A might be a biomarker of poor prognosis for breast cancer.

**Table 1:** Clinical characteristics of breast cancer patients.

Characteristic	Low expression of TIMM8A	High expression of TIMM8A	p
n	541	542	
T stage, n (%)			0.016
T1	155 (14.4%)	122 (11.3%)	
T2	295 (27.3%)	334 (30.9%)	
T3	77 (7.1%)	62 (5.7%)	
T4	13 (1.2%)	22 (2%)	
N stage, n (%)			0.455
N0	258 (24.2%)	256 (24.1%)	
N1	185 (17.4%)	173 (16.3%)	
N2	50 (4.7%)	66 (6.2%)	
N3	38 (3.6%)	38 (3.6%)	
M stage, n (%)			0.162
M0	436 (47.3%)	466 (50.5%)	
M1	6 (0.7%)	14 (1.5%)	
PR status, n (%)			< 0.001
Negative	114 (11%)	228 (22.1%)	
Indeterminate	2 (0.2%)	2 (0.2%)	
Positive	402 (38.9%)	286 (27.7%)	
ER status, n (%)			< 0.001
Negative	61 (5.9%)	179 (17.3%)	
Indeterminate	0 (0%)	2 (0.2%)	
Positive	457 (44.2%)	336 (32.5%)	
HER2 status, n (%)			0.014
Negative	292 (40.2%)	266 (36.6%)	
Indeterminate	4 (0.6%)	8 (1.1%)	
Positive	63 (8.7%)	94 (12.9%)	
Age, n (%)			0.120
<=60	287 (26.5%)	314 (29%)	
>60	254 (23.5%)	228 (21.1%)	
OS event, n (%)			< 0.001
Alive	485 (44.8%)	446 (41.2%)	
Dead	56 (5.2%)	96 (8.9%)	

### Mutations in TIMM8A in BC

**Figure 5:**
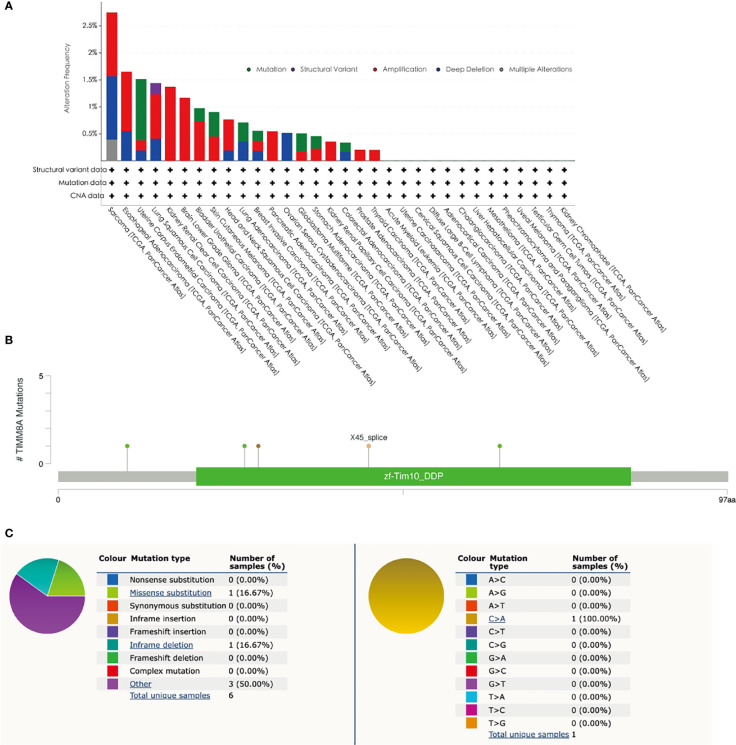
Mutations in BC. **(A)** The gene alteration of TIMM8A in pan-cancer. **(B)** The schematic representation of TIMM8A mutations in BC (cBioPortal). **(C)** The mutation types in the Catalogue of Somatic Mutations in Cancer (COSMIC) database.

The mutation frequency of TIMM8A in breast cancer was evaluated in the cBioPortal database. The selection included an analysis of 1084 samples. The somatic mutation frequency of TIMM8A in breast cancer was 0.55% and was mainly composed of missense mutations (**Figure 5A**). The type, and sites of TIMM8A genetic alterations were further shown in **Figure 5B**. In addition, the mutation type of TIMM8A was further evaluated in another database, COSMIC. For clarity, **Figure 5C** show a pie chart of two types of mutations. Missense substitution occurred in approximately 16.67% of the samples and in-frame deletion occurred in 16.67% of the samples. Substitution mutations mainly occurred in C > A 1 (100%).

### TIMM8A methylation in BC patients

**Figure 6:**
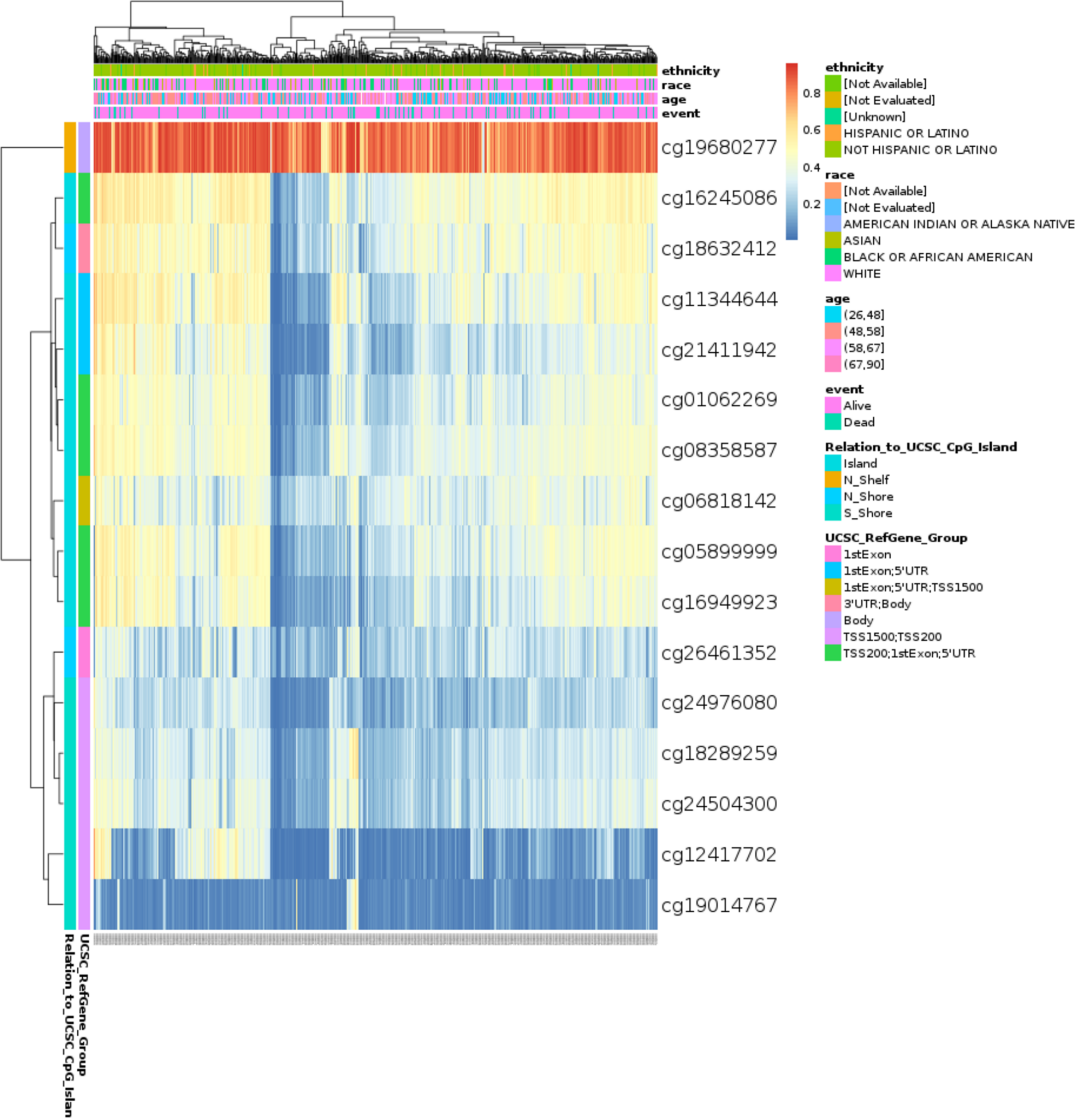
Visualization between methylation levels and TIMM8A expression.

The DNA methylation level of TIMM8A and the prognostic value of each CpG were studied using the MethSurv tool. MethSurv results suggest 16 methylated CpG check points, of which cg19680277 had the highest DNA methylation (**Figure 6**). Methylation levels at seven CpG check points were associated with prognosis, namely cg01062269, cg24976080, cg19680277, cg21411942, cg19014767, cg16245086, and cg08358587 (*p* < 0.05) (**Table 2**). The overall survival rate of patients with high TIMM8A methylation at these CpG check points was lower than that of patients with low TIMM8A methylation.

**Table 2:** Effect of methylation level on prognosis of BC.

Name	HR	CI	P.value
cg18289259	0.783	(0.531;1.155)	0.217493894
cg06818142	0.74	(0.503;1.09)	0.127974278
cg11344644	0.716	(0.471;1.088)	0.117981842
cg24504300	0.726	(0.492;1.071)	0.106309649
cg12417702	0.652	(0.396;1.074)	0.093175667
cg26461352	1.537	(0.931;2.539)	0.092862056
cg16949923	0.713	(0.482;1.056)	0.091046689
cg05899999	0.692	(0.467;1.024)	0.065242539
cg18632412	0.634	(0.392;1.025)	0.063185771
cg01062269	0.664	(0.449;0.981)	0.039517476
cg24976080	0.657	(0.446;0.97)	0.034368727
cg19680277	0.63	(0.428;0.928)	0.019267915
cg21411942	0.584	(0.388;0.878)	0.009735486
cg19014767	0.554	(0.364;0.845)	0.006088526
cg16245086	0.563	(0.382;0.829)	0.003579663
cg08358587	0.519	(0.348;0.775)	0.00135529

### Differential genes associated with TIMM8A in breast cancer

**Figure 7:**
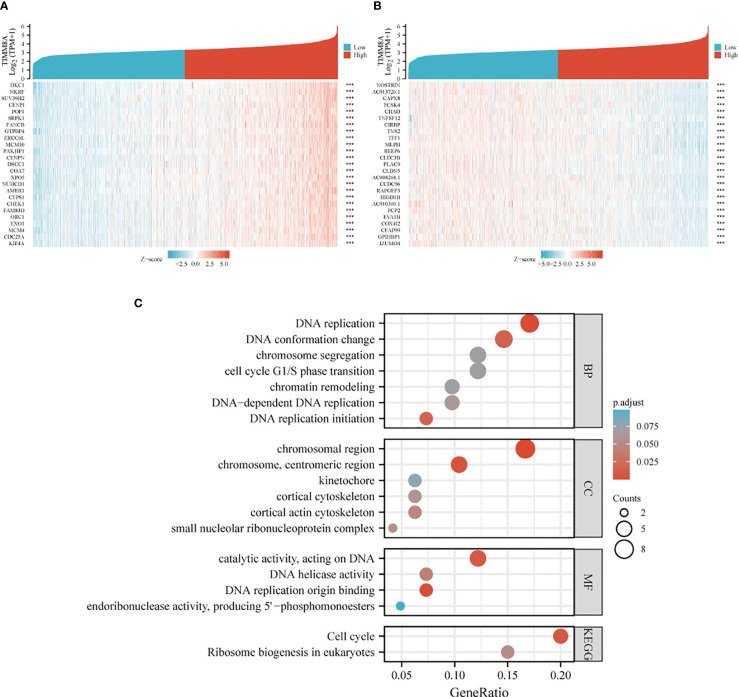
Heat map and enrichment of TIMM8A-related differentially expressed genes in breast cancer. **(A)** heat map shows genes positively correlated to TIMM8A (showing the first 25 genes). **(B)** heat map shows genes negatively correlated with TIMM8A (showing the first 25 genes). **(C)** GO analysis and KEGG enrichment of differentially related genes. (***p > 0.001).

TIMM8A-related differentially expressed genes in breast cancer were identified according to the TCGA database. Differentially expressed genes related to TIMM8A were identified based on the Spearman test. The first 25 positive (r > 0) and the first 25 negative (r < 0) related genes were shown in the heat map (**Figure 7A, B**). According to Spearman’s test results, positively correlated genes with coefficients > 0.3 were selected. The results showed that 2518 genes were positively correlated with TIMM8A, and 159 were negatively correlated. As shown in **Figure 7C**, these genes were selected for visualization and analysis. GO and KEGG enrichment analysis was performed on the differentially expressed genes. The following biological processes were significantly affected: DNA replication, DNA replication initiation, DNA conformation change, DNA-dependent DNA replication, cell cycle G1/S phase transition, chromosome segregation, chromatin remodeling, etc. The terms of cell components are mainly enriched in chromosomal region, chromosome, centromeric region, cortical actin cytoskeleton, small nucleolar ribonucleoprotein complex, cortical cytoskeleton, kinetochore, etc. Molecular functional terms mainly focus on DNA replication origin binding, catalytic activity, acting on DNA, DNA helicase activity, endoribonuclease activity, producing 5’-phosphomonoesters, etc. KEGG results show that co-expressed genes are mainly involved in Cell cycle, Ribosome biogenesis in eukaryotes pathway.

### Single gene difference analysis Gene Set Enrichment Analysis (GSEA)

**Figure 8:**
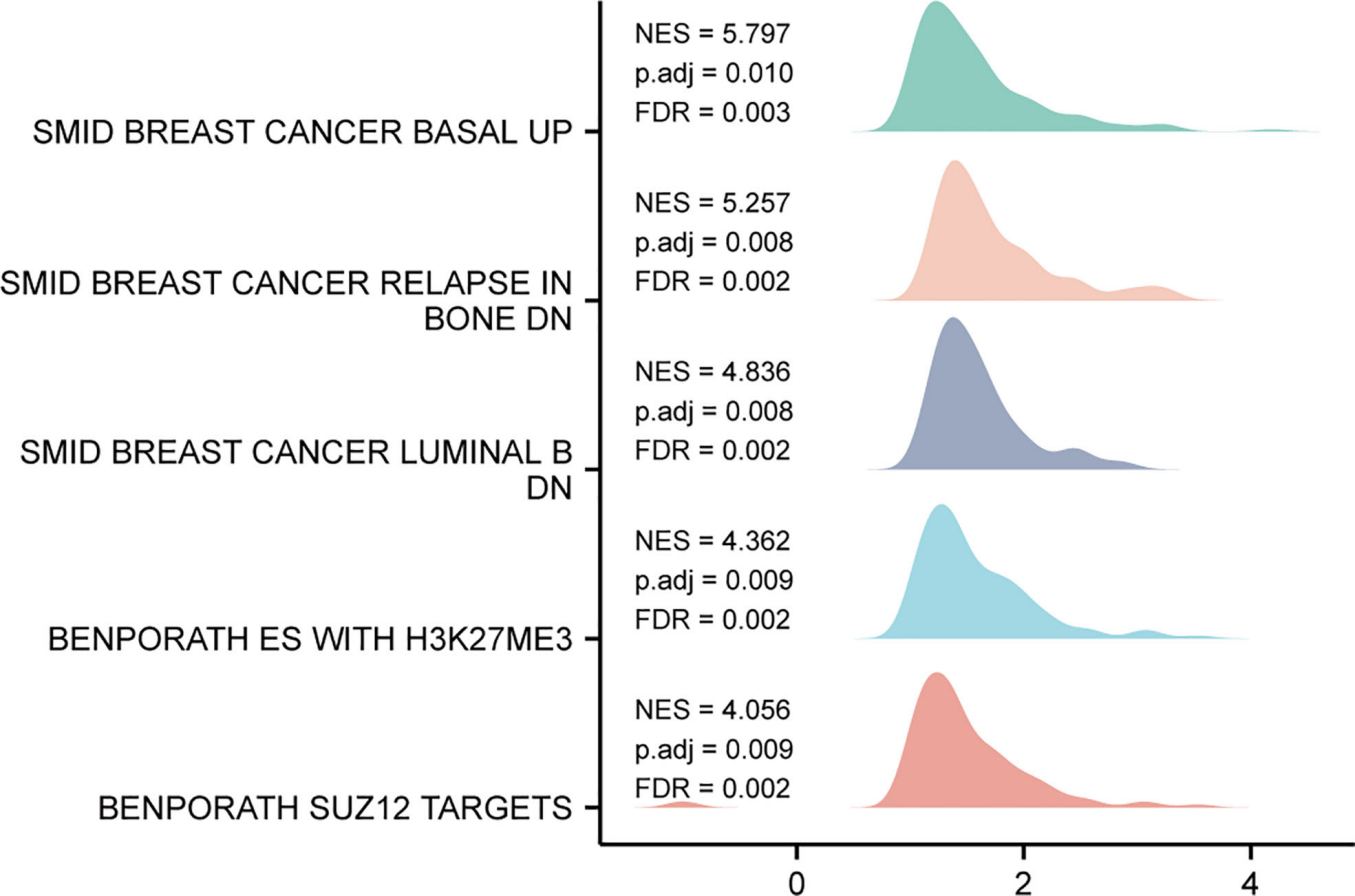
GSEA analysis results. Genes enriched in representative pathways by GSEA functional analysis.

Based on the median expression value of TIMM8A, we analyzed the DEGs between the TIMM8A low-expression group and the high-expression group in order to understand the biological function of TIMM8A and GSEA pathway analysis (**Figure 8**). The results show that TIMM8A is rich in BREAST_CANCER_BASAL_UP, BREAST_CANCER_RELAPSE_IN, BONE_DN, BENPORATH_ES_WITH_H3K27ME3, BENPORATH_SUZ12_TARGETS.

### Univariate and multivariate cox regression analysis of TIMM8A and clinical data

Univariate and multivariate survival analyses were performed using Cox regression models. Multivariate Cox analysis compared the effects of TIMM8A expression and other clinical features on survival. TIMM8A was separated into two groups based on the best cut-off value: high expression and low expression. The statistical significance of two tailed test was set as 0.05.

OS was linked with age, T stage, N stage, M stage, HR status, and TIMM8A expression in a univariate Cox regression analysis. In addition, multivariate Cox regression analysis showed that TIMM8A expression (HR=1.688, p=0.016 is an independent prognostic factor for BC patients (**Table 3**). T stage, N stage, M stage, and TIMM8A expression were all linked to OS in a univariate Cox regression analysis. These findings demonstrate that TIMM8A is upregulated in BC and is linked to a negative prognosis.

**Table 3:** Univariate and Multivariate Cox Regression Analysis of TIMM8A and Clinical Data.

Characteristics	Total(N)	HR(95% CI) Univariate analysis		P value Univariate analysis	Characteristics	Total(N)	HR(95% CI) Multivariate analysis		P value Multivariate analysis
T stage	1079		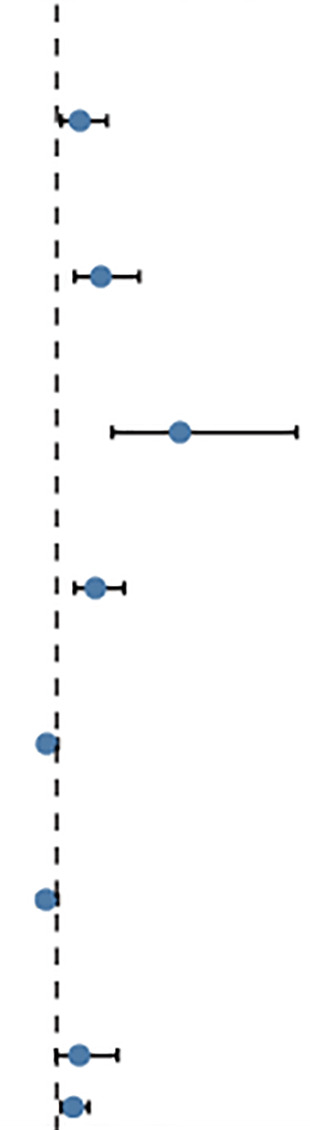		T stage	1079		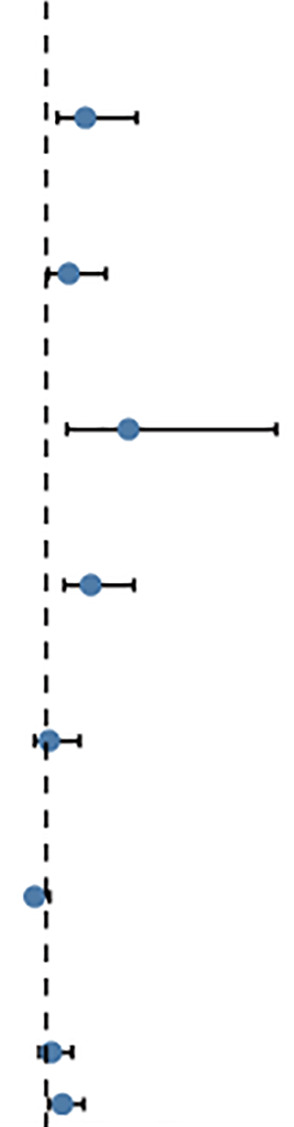	
T1&T2	905	Reference			T1&T2	905			
T3&T4	174	1.608 (1.110-2.329)		0.012	T3&T4	174	2.66 (1.469-4.834)		0.001
N stage	1063				N stage	1063			
N0&N1	871	Reference			N0&N1	871			
N2&N3	192	2.163 (1.472-3.180)		<0.001	N2&N3	192	1.960 (1.092-3.520)		0.024
M stage	922				M stage	922			
M0	902	Reference			M0	902			
M1	20	4.254 (2.468-7.334)		<0.001	M1	20	4.492 (1.872-10.776)		<0.001
Age	1082				Age	1082			
<=60	601	Reference			<=60	601			
>60	481	2.020 (1.465-2.784)		<0.001	>60	481	2.892 (1.773-4.717)		<0.001
PR status	1029				PR status	1029			
Negative	342	Reference			Negative	342			
Positive	687	0.732 (0.523-1.024)		0.068	Positive	687	1.122 (0.520-2.422)		0.77
ER status	1032				ER status	1032			
Negative	240	Reference			Negative	240			
Positive	792	0.712 (0.495-1.023)		0.066	Positive	792	0.505 (0.224-1.135)		0.98
HER2 status	715				HER2 status	715			
Negative	558	Reference			Negative	558			
Positive	157	1.593 (0.973-2.609)		0.064	Positive	157	1.215 (0.698-2.117)		0.491
TMM8A	1082	1.438 (1.22-1.844)		0.004	TMM8A	1082	1.688 (1.100-2.588)		0.016
			2 4 6					0 3 69	

### Construction and evaluation of the nomogram

**Figure 9:**
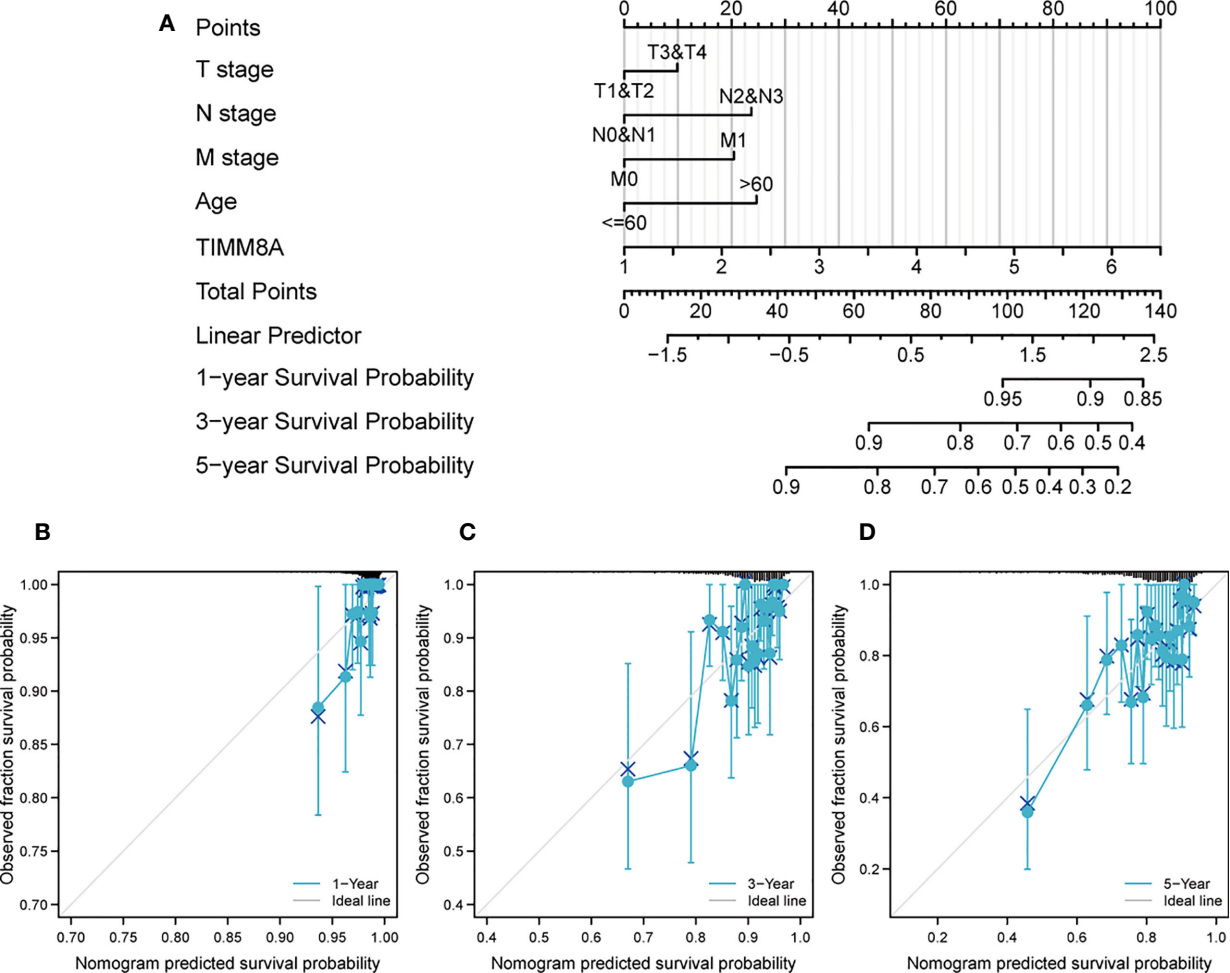
Construct a Nomogram to predict the survival probability of BC patients. **(A)** Nomogram predicts the 1-year, 3-year, and 5-year BC survival probability composed of TIMM8A and independent clinical risk factors. **(B–D)** Calibration plot of nomogram for predicting probabilities of 1-, 3-, and 5-year survival probability. Grey line indicates actual survival.

We created a Nomogram of TIMM8A and independent clinical risk indicators (T/N/M staging, TIMM8A, age) to give a quantitative way for predicting the prognosis of BC patients (**Figure 9A**). A point scale was used to award points to these variables in this Nomogram based on multivariate cox analysis. Draw a straight line upward to establish the variable’s number of points, and then modify the total of the points allotted to each variable to a range of 0 to 100.The integrals of the variables are summed together to obtain a total score. BC patients’ 1-year, 3-year, and 5-year survival probabilities were calculated vertically from the total point axis to the outcome axis (**Figures 9B-D**). The nomogram’s C-index for OS prediction was 0.775. (0.745-0.805).

### The correlation between TIMM8A and immune cell infiltration

**Figure 10:**
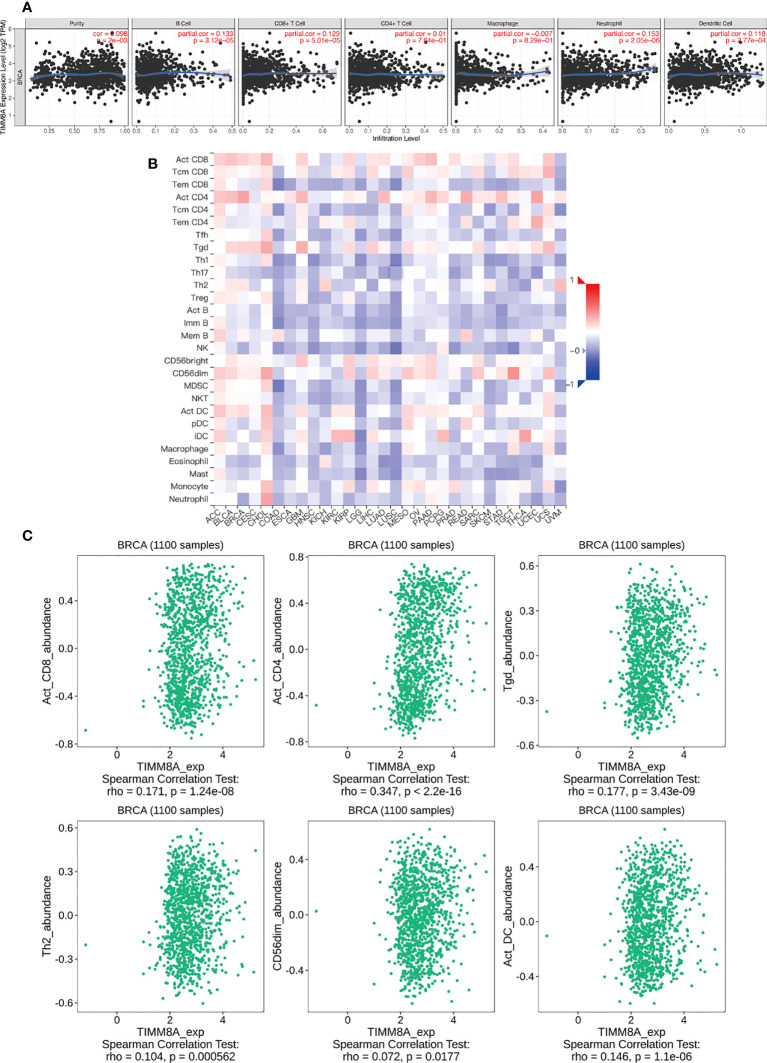
TIMM8A expression correlates with immune level. **(A)** TIMM8A expression in breast cancer correlates with tumor purity, B cells, CD4 + T cells, CD8 + T cells, macrophages, neutrophils, and dendritic cells. **(B)** TIMM8A expression in human tumors correlates with 28 lymphocytes. **(C)** Relationship between TIMM8A and CD8 + T cells, CD4 cells, Tgd cells, Th2 cells, CD56 cells, DC cells.

We analyzed the correlation between TIMM8A expression and 6 tumor-infiltrating immune cells in the TIMER database. As shown in **Figure 10A**, the expression of TIMM8A is related to tumor purity (r = 0.098, P = 2e − 03), B cells (r = 0.133, P = 3.12e − 05), CD8 + T cells (r = 0.129, P = 5.01e − 05), CD4 + T cells (r = 0.01, 7.64e-01), macrophages (r = -0.007, P = 8.29e − 01), and neutrophils (r = 0.153, P = 2. 05e − 06), dendritic cells (r = 0.118, P = 2.77e − 04). **Figure 10B** depicts the TISIDB database’s link between TIMM8A expression and 28 lymphocytes. **Figure 10C** show that the expression of TIMM8A is related to the abundance of CD8 + T cells (r = 0.171, P = 1.24e-08), CD4 cells (r = 0.347, P < 2.2E-16), Tgd cells (r = 0.177, P = 3.43-09), Th2 cells (r = 0.104, P = 0.0000562), CD56 cells (r=0.072, P = 0.0177), and DC cells (r = 0.146, P = 1.1e-06). These findings implied that TIMM8A may play a unique function in the immunological invasion of breast cancer.

### Relationship between TIMM8A expression and immune checkpoints in BC

**Figure 11:**
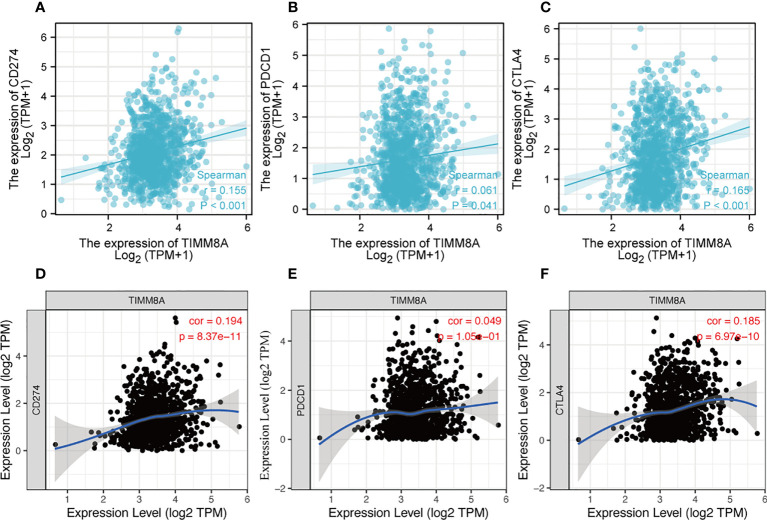
Correlation between TIMM8A and PDL-1 (CD247), PD-1, CTLA-4 expression in BC. The TIMER and TCGA database respectively TIMM8A expression and PDL-1 **(A, D)**, PD-1 **(B, E)** and CTLA 4 **(C, F)**.

PDL-1 (CD274), PD-1 (PDCD-1) and CTLA-4 were key immune checkpoints involved in tumor immune escape. Given TIMM8A’s putative oncogenic function in BC, the association of TIMM8A with PDL-1, PD-1, and CTLA-4 was investigated in both the TIMER and TCGA databases. In BC, there was a substantial positive connection between TIMM8A and PDL-1 and CTLA-4 (**Figure 11**).

### Small molecular drugs

**Figure 12:**
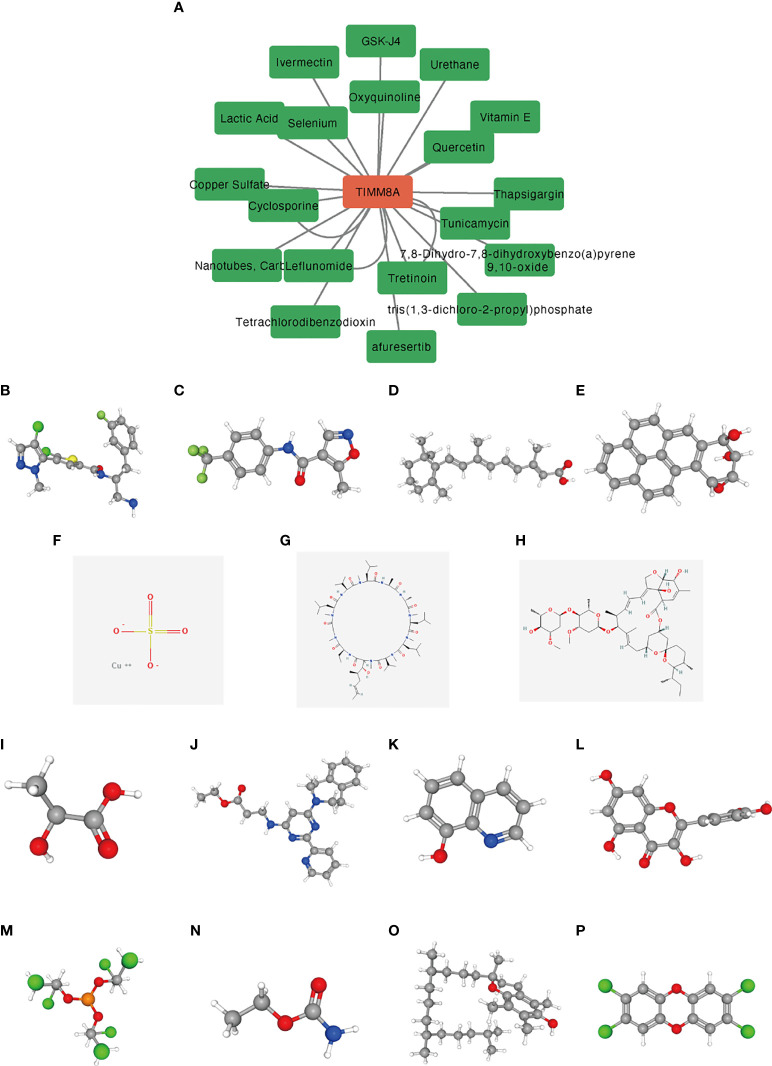
Predicting potential drugs and molecular structures that affect TIMM8A. **(A)** CTD database predicts potential drugs that affect TIMM8A, and green represents drug molecules that inhibit TIMM8A. **(B–P)** PubChem database predicts molecular structures of 15 targeted drugs.

We used the CTD database to analyze the correlation between TIMM8A and potential drugs (**Supplementary Table 1**). A total of 15 drugs were identified as having inhibitory effects on TIMM8A (**Figure 12**). These drugs had potential inhibitory effects on TIMM8A.

## Discussion

TIMM8A is a member of zinc finger proteins family that form hetero-oligomeric complexes in the mitochondrial intermembrane region. TIMM8A mutations are linked to the Mohr-Tranebjaerg syndrome, a progressive neurological illness (16). TIMM8A has been linked to the growth of SKOV3/DDP ovarian cancer cell line subcutaneous xenografts (11). However, the mRNA level of TIMM8A and its predictive significance in BC have not been well explored. Breast cancer systemic therapy choices contains surgery, endocrine treatment, radiation treatment, and targeted treatment. In the management of hormone receptor-positive individuals, endocrine therapy is extremely important. According to certain accounts, various hormone levels alter a patient’s prognosis (17–19). We found that upregulation of TIMM8A was inversely correlated with hormone receptor status. Furthermore, because ER and PR status are linked to prognosis, TIMM8A might be used to predict hormone receptor status and the requirement for endocrine treatment. Those with high mRNA expression in breast cancer had a poorer survival rate than patients with low TIMM8A levels. TIMM8A expression exhibited an excellent capacity to differentiate cancer and normal tissues, suggesting that it could be took as a potentially beneficial diagnostic and prognostic marker for BC.

Even if gene mutations were linked with bad prognosis and were closely related to malignancies, the rate of TIMM8A gene alterations in BC was only approximately 0.18%, and there was no substantial association between gene changes and poor OS. DNA methylation was a ubiquitous epigenetic process seen in all cancers (20, 21). We investigated the link between the degree of DNA methylation in TIMM8A and the prognosis of cancer patients. Hypomethylation was associated with prognosis at seven CpG check points, including cg19014767, cg16245086 and cg08358587.

Immunotherapy had drastically transformed the paradigm of cancer treatment in recent decades and had been acknowledged as a potential therapeutic frontier (22, 23). Furthermore, mounting data suggested that the TIME was linked to tumor growth and metastasis (7). Furthermore, several studies had demonstrated that series of immune cells were related with BC malignancies (24). In addition, the effectiveness of immunotherapy not only required sufficient immune cells penetration into the tumor microenvironment, but also relied on the adequate expression of IC. There had been no research on the relationship between TIMM8A expression and immune cells in BC. TIMER was employed in this work to discover that this gene might enhance the invading immune cells such as CD8 + T cells, but it was also favorably connected with the expression of PD-L1 and CTLA-4. As a result, this gene might enhanced PD-L1 expression, which resulting in an immune microenvironment suppressive state. It suggested that targeting TIMM8A might promote the efficacy of ICIs in BC. As a result, more research into its mechanism was required in order to eliminate the immunosuppressive impact of TIMM8A and enhance the OS of tumor patients.

15 medicines were found by utilizing the CTD database to forecast medications which inhibited TIMM8A.TIMM8A may be targeted and was predicted to bring new advances in BC therapy after being utilized in the PubChem database to establish the molecular structure of discovered medicines.

We also discovered that TIMM8A mRNA and protein expression are up-regulated in BC tissues in our study. According to ROC curve study, TIMM8A might considered as a potential diagnostic biomarker to distinguish BC from normal tissues. We verified that TIMM8A expression was linked with short OS using the Kaplan Meier curve and univariate analysis, and TIMM8A could be employed as a possible biomarker for poor prognosis of BC. Furthermore, TIMM8A might have a role in the immune escape of BC.

At present, this study still had some limitations. Firstly, the expression of TIMM8A and its prognostic significance were examined through online database. We required further studies on *in vitro*/animal experiments, and clinical samples validations. Secondly, mechanism experiments in both vitro and *in vivo* should be designed to investigate how TIMM8A affected BC immune invasion.

To summarize, we firstly discovered in this work that TIMM8A was greatly up-regulated in BC. TIMM8A could considered as a prognostic candidate marker, and that it might play a special function associated with immune infiltration.

## Conclusions

In conclusion, TIMM8A was important and could be used as a potential biomarker for prognosis in BC. Furthermore, TIMM8A could have a role in the immunological invasion of BC.

## Data availability statement

The original contributions presented in the study are included in the article/**Supplementary Material**. Further inquiries can be directed to the corresponding authors.

## Author contributions

WY and JZ conceived and designed the study. YZ and LL performed the experiments. YW and PB analyzed the data. YZ wrote the manuscript. All authors contributed to the article and approved the submitted version

## Conflict of interest

The authors declare that the research was conducted in the absence of any commercial or financial relationships that could be construed as a potential conflict of interest.

## Publisher’s note

All claims expressed in this article are solely those of the authors and do not necessarily represent those of their affiliated organizations, or those of the publisher, the editors and the reviewers. Any product that may be evaluated in this article, or claim that may be made by its manufacturer, is not guaranteed or endorsed by the publisher.
